# Green shared vision: A bridge between responsible leadership and green behavior under individual green values

**DOI:** 10.1016/j.heliyon.2023.e21511

**Published:** 2023-10-24

**Authors:** Nimra Younas, Md Billal Hossain, Aleena Syed, Sarmad Ejaz, Faisal Ejaz, Tahir Saeed Jagirani, Anna Dunay

**Affiliations:** a Hailey College of Banking and Finance, University of the Punjab, Lahore, Pakistan; b Business Management and Marketing Department, School of Business and Economics, Westminster International University in Tashkent (WIUT), Tashkent, 100047, Uzbekistan; c Department of Management Sciences, University of Okara, Okara, 56300, Pakistan; d School of International Relations, Minhaj University, Lahore, 54770, Pakistan; e School of Economics, Finance and Banking, University Utara Malaysia (UUM), Malaysia; f John von Neumann University, 1117 Budapest, Hungary

**Keywords:** Responsible leadership, Green shared vision, Individual green values, Green behaviors

## Abstract

The pressure on businesses to be environmentally conscious and focus on sustainable development is accruing due to environmental challenges. Companies are adopting ecological practices and policies to improve their environmentally friendly performance. To achieve this, organizations must substantiate and change the behavior of workers to align their behavior with the organization's ecological objectives. The study endeavors to integrate research on the responsible style of leaders and green behaviors of employees (in-role and extra-role green behaviors) through the mediation of green shared vision and analyze the moderation mechanism of individual green values. For collecting the data, a questionnaire-based survey was conducted among MBA executive program students with at least a year of experience in manufacturing. Out of the 450 questionnaires distributed, only 307 useful responses were obtained. The collected data has been analyzed using SPSS and AMOS. Ethical standards were followed, and participants were assured that their responses would be confidential. The study found that responsible leadership positively impacts green behaviors among employees. This means that when leaders within an organization demonstrate responsible and environmentally conscious behavior, it tends to encourage employees to engage in green behaviors. The study also discovered that a “green shared vision” partially mediates the relationship between responsible leadership and in-role green behavior. In contrast, green shared vision does not mediate the relation between responsible leadership and extra role green behavior. Moreover, this study also finds that the relationship between green shared vision and in-role and extra-role green behavior is strengthened when individual green values moderate it. The study highlights the importance of responsible leadership and the role of green shared values and individual green values in promoting environmentally friendly behavior in the workplace.

## 1 Introduction

Environmental challenges, including climate change and resource depletion, have intensified recently, creating significant pressure on businesses worldwide [1]. Companies are increasingly expected to develop environmental consciousness and prioritize sustainable development as part of their corporate responsibility [2]. While regulations and rules are essential, a crucial aspect of an organization's green performance relies on its employees' positive responses and actions concerning environmental concerns. Employees act as the driving force in implementing environmentally friendly policies and practices within the company [3]. Green behaviors include a wide range of actions and conduct employees undertake to enhance the organization's sustainability [4]. These include in-role behaviors, which are directly related to an employee's job, and extra-role behaviors, which go beyond job requirements [5].

In contrast, employees’ ecological behavior contribution remains open to discussion [6]. Evidence suggests that such behaviors can lead to reduced energy costs [7], increased profits [8], improved reputation, competitive advantage, and long-term progress [9]. Recognizing the significant macro-level impact of employee ecological behavior on sustainability, fostering such behavior at various organizational levels is crucial, with leadership playing a pivotal role [10,11].

There are different predictors at an individual level [12] and business levels [13]. In organizational-level predictors, the role played by the leaders to enhance the efficiency of ecological initiatives cannot be overlooked [14]. Within the literature, the leadership style has been considered an important antecedent since the leaders work as the agents of the organization to treat the workers by exerting significant influence on the personnel employees [15]. Numerous leadership styles have a diverse effect on the consequences based on the environment [6]. The influence of green transformational leaders, despotic leaders, inclusive leaders, and ethical leaders on the environmental behaviors of employees has been investigated by research numerous research studies [16].

However, the study highlights that the influence of responsible leadership style on employees' green behaviors has not been adequately explored [17]. The responsible style of leader is proposed to be a phenomenon that is ethical, relational, and social, occurring in the social practices of relations for sustainable value creation [17]. Responsible leaders proactively promote the ecology within their organization [18]. Numerous research studies explained the direct association between responsible leadership and green behavior [19], and some researchers propose that moderators and mediators in this relationship are essential [20], warranting further investigation.

The research on green shared vision [21] is referred to as developing a mutual vision aiming at sustainable and ecological development. The company engages in ecological conduct by sharing mutual ideas with the workers [22]. Leaders of a company are an essential element in the development of a vision of an organization. Vision is created by the supervisor's behavior [23]. Still, the researchers do not inspect the relationship between the responsible style of leaders and green shared values [24]. Since responsible leaders consider the different stakeholders' interests, they also try to balance the business's and stakeholders' interests using other management procedures [25]. The green shared vision of an organization can be fostered by the responsible leadership style [24].

Along with this, responsible leaders emphasize improving the environment [26], which results in cultivating a green shared vision through making mutual plans to protect the environment. Heedlessly of the significant role of the behavior of employees to improve environment-related outcomes, there has been seen little empirical evidence of research that associate the approaches of leaders to the environmentally friendly behaviors of the employees through the mediating role of green shared vision [27].

The joint influence of social and organizational context on individual differences activates ecological behavior [28]. The individual's environmentally friendly values positively depict an individual's behaviors that are also directed toward going green [29]. Employees align their values and identifications by connecting with the company, increasing their commitment to accomplishing the organization's objectives [30]. The workers' ecological beliefs interactively influence the environment-friendly behavior of workers in the organization [31]. The green shared vision of the company indicates the consequences of the employee judgment about the ecological values in the presence of which the worker's green behaviors can be heightened. The literature review provides no evidence about the moderating mechanism of the green values of individuals in relation to green shared vision and green behaviors (in-role and extra-role green behaviors).

The study contributes to the existing body of knowledge about the organization's leaders, individuals, or employees' ecological behaviors and values. First, the study is significant from the leadership perspective, as it examines the direct relation among responsible leaders and in-role ecological behaviors and the employees' extra role environmental friendly behaviors. Such an investigation offers valuable understanding and insight into the role of leaders and its importance in encouraging eco-friendly behaviors in employees while improving the organization's environmentalism concept. Secondly, the research focuses on combining the perspective of the leader, which is considered to be an organizational level predictor, as well as the process that is employee-centered, taken up as an individual level predictor, in the context of supplies and values fit (S–V fit) theory to more comprehensively examine the association of responsible leaders and in-role eco-friendly behaviors and extra-role environmental behaviors of personnel. Even extant literature examined the relationship, but there is a contradiction in the research findings [32]. Thirdly, building on the S–V fit theory [33], the study will enrich the literature on the employees' environmental friendly behaviors by adding green shared vision as a supplementary mechanism, which mediates the relationship between responsible leaders and eco-friendly behaviors of the workers. In this regard, by exploring the literature, it seems that there is a deficiency of literature providing empirical evidence of the research that links the leadership approach of responsible leadership with the eco-friendly behavior of employees via the mediating role of green shared vision [34]. Besides this, the study contributes to the literature by repossessing theoretical provisions from the S–V fit theory for examining the individual green values as a condition variable among the association of green shared vision and employee ecofriendly in-role and extra-role green behaviors of the employees. The aim of the present study is to explain responsible leadership and green behaviors (in-role green behaviors and extra-role both) through the mediator green shared vision and moderator individual green values. The present study is based on following objectives;

RO1 To identify the impact of responsible leaders on employee in-role and extra-role green behaviors.

RO2 To analyze the mediating role of green shared vision among the relationship of responsible leadership and in-role and extra-role green behaviors.

RO3 To examine the moderating mechanism of individual green values among the link of green shared vision and in-role and extra-role green behaviors.

The rest of the paper has the following structure. Section 2 describes the literature review of individual variables and hypotheses development. Theoretical framework and conceptual model have been explained in section 2. In section 3 procedure, sample, and data collection, and section 4 about results. While section 5 explain discussion about the findings of this study. Section 6 is related to implications and 7 related to limitation and future directions.

## 2 Literature review

### 2.1 Variables of research

#### 2.1.1 Responsible leadership

The supervisor must value the company's goals for sustainable development, which consider recognizing the social responsibility towards the swelling challenges of the safety of food, pollution, and wastage of resources, which are subject to responsible leadership [35]. Responsible leadership includes three main elements: effectiveness, ethics, and sustainability. Responsible leaders act ethically and set examples for their employees [36]. Responsible leaders do things in the right way so that the employees also follow the path of their leaders [37]. Keeping in view the perspective of [38], responsible leaders constrain the followers' unethical behaviors.

Responsible leadership emphasizes responsibility matters, such as building trust, making choices for green actions, morally making decisions, and ecological development [38]. According to Ref. [17] research study, responsible leaders are the one who creates and maintains trust relations with the stakeholders within and external of the business to fulfill societal obligations. It also includes the ethical, interactive, and social interfaces established and sustained among individuals who influence and individuals influenced by the company's specific practices [39]. Accordingly [25], explained the term responsible leadership as an amalgamation of social consciousness, ethics, commitment of stakeholders in the practices of the organization, and leadership.

#### 2.1.2 Green behaviors

Individual green behaviors are crucial to explaining the company's sustainability plan in practical results. It is considered the foundation aimed at the ecological improvement of the business [40]. The primary aim of green behaviors is to protect the environment or for minimizing the damage for the environs [41], which consists of both the dimensions of role as well as different roles. The in-role green behaviors of workers and the extra-role green behaviors collectively create values for business firms [42]. The classification of green behaviors is based on the expectations of the organizations from their employees [43]. The policies of the business firm regarding the disposal of hazardous materials or protection of water from venomous constituents, ultimatum for green behaviors from personnel. The behaviors anticipated by the employees in the organization are usually included in the formal job descriptions of the workers (that is, in-role green behaviors), however, in the job description of employees, the extra-role behaviors are not mentioned since these behaviors are considered to be clear as suggestions for the employees for protecting the environment [44]. Extra role behaviors may include switching off computers, lights, and fans when the employees are not using these things or tuning these off when the employees are supposed to leave the workplace for any engagement [44]. Undeniably, both employee in-role ecological and extra-role environmentally friendly behaviors are required to align the green strategies of the company with its green goals [45], nevertheless, the exhibition of green behaviors are subject to different situations as the workers have diverse levels of discretion [46] (see Table 1).

**Table 1:** Review of literature.

Study	Context	Predictors	Outcomes	Findings
[47]	High Tech Industry	Green Human Resource Management(GHRM) and Psychological Mechanism	Green Behavior	Green human resource management significantly positivity effect on Green Behavior and psychological mechanism mediates between them.
[48]	services and manufacturing sectors	Responsible Leadership, Green shared Visions, Organizational commitment,	Pro environmental behavior	The authors of this study proposed two mechanisms organizational commitment and a green shared vision to explain the connection between ethical management and eco-friendly actions taken by workers. It was shown that employees having a higher internal environmental locus of control were more likely to benefit from the indirect influence of responsible leadership on pro-environmental behavior brought about by a green common vision.
[49]	Five star Hotels in China	Corporate Social Responsibility, Green Behavior, Responsible Leadership and GHRM	Employees Task performance	Green behavior mediates between corporate social responsibility and employees task performance. The results also suggest that GHRM moderates between CSR-GHRM. Moreover responsible leadership moderates to GHRM.
[13]	A family holding company with eight diversified energy sectors	RL, Psychological Ownership	Employees Green Behavior	Responsible leadership impact of employee's green behavior through psychological ownership.
[50]	Manufacturing Companies	Top Management responsible Leadership, GHRM, Environmental felt responsibility	Organization Citizenship Behavior	TMT responsible leadership significant impact on green human resource management and green human resource management and environmental felt responsibility mediates.

#### 2.1.3 Green shared vision

The common vision developed in the organization that is directed towards ecological and environmental development is considered the green shared vision [51]. Shared vision exists in the company when the supervisors communicate the organization's aims to their subordinates to accomplish the company's aims [52]. Whereas green shared vision indicates a direction that is strategic, common, and clear to accomplish the shared purposes of the business along with accomplishing the aspirations that the company's employees have internalized [53]. Green shared vision provides proper guidelines to the organization's employees to overcome challenges as well as performing tasks that are related with work [51]. The workers employed in a business firm are usually inclined towards forming unswerving green vision discernment since they are interacting, communicating, and working in that environs [51]. The companies are engaging themselves in ecological practices by means of sharing a mutual vision with workers [22], along with giving meaning to the everyday activities of employees at work.

#### 2.1.4 Individual green values

For achieving the aspirations of the business firm, the individuals as employees play a significant role. Likewise, ecological practices can be effectively implemented when the green values of the individuals are developed [54]. Individual values help explain the employees' behaviors and attitudes [55]. Implementing a company's rules and policies significantly influences employees' behavior [56]. Similarly, the study of [43] suggested that green values of the individuals are affected by ecological practices. It indicates that the ecological methods and practices of the organization enhance the environmentally friendly values of the individual [57]. The green values individuals possess as employees are crucial in their understanding of the role of becoming environmentally conscious and vitality to focus on becoming greener at work [58].

### 2.2 Theoretical framework

**Fig. 1:**
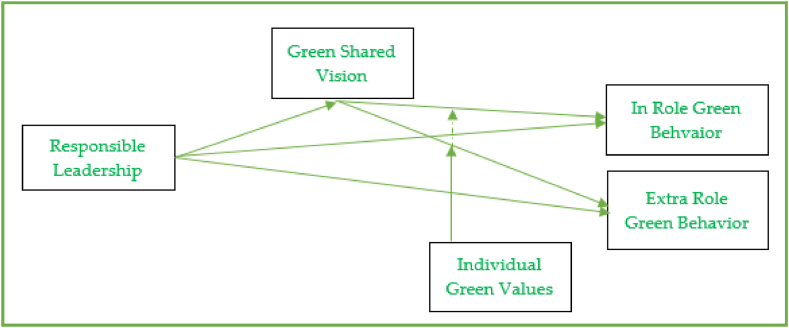
Conceptual framework: Author's creation.

**Fig. 2:**
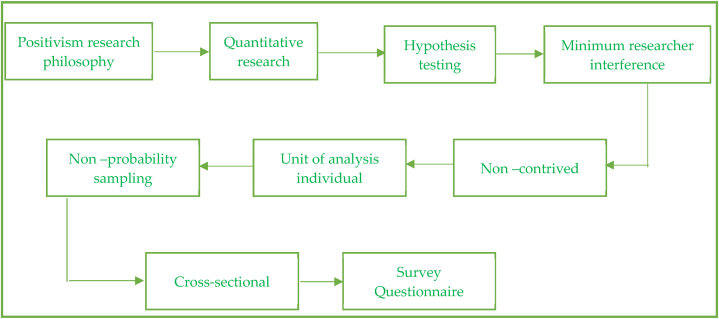
Graphical representation of Research design.

The present study is grounded on the supplies-values fit (S–V fit) theory to retrieve theoretical support from the theory [33]. The S–V fit theory claims that the company's individuals value the supplies provided to them by the organization [33]. So the supplies of the organization and valuing of employees is considered a supplemental fit when the individuals of the organization believe that their objectives and aims are unswerving with the values and goals of their company [59]. For this reason, relying on the theory of supply-value fit, the existing study argues that responsible leaders are considered to be the supplies provided to the individuals from the organization, and the individuals value the behaviors of their leaders. Consequently, the workers imitate the ecological behaviors of leaders and try to improve the work environment. The research studies also highlight that the style of leadership and the environmental friendly behaviors of the employees are closely related conceptions [60]. The eco-friendly behaviors of the supervisor and subordinates are positively significant [61] since the manager's behaviors exhibit their beliefs, and their values are passed on to their employees by role models. In the presence of responsible leadership, the subordinates understand and recognize the prominence of their ecological behaviors [41]. Responsible leaders pass signals to employees by showing their concerns about social responsibility and the environment, which creates awareness in the employees to behave responsibly [62]. The study assumes that the responsible leadership style unswervingly and positively affects worker in-role environmental behaviors and their extra-role ecological behaviors. Reclaiming support from the theory of S–V fit [33], the theory presumes that when supplies are provided to the recipients by the organization, they evaluate these supplies with the values, so when values are overweighing by the supplies, the recipients (employees) feel fit [33]. The feeling of supplies overweighing the values intensifies the fit perception among the objectives of employees and business firm [59]. The theory also suggests that when the supplies offered by the company are at the same level or exceed the level of individual values, a situation of fit arises, leading towards positive employee behaviors and attitudes. So considering the S–V fit theory, the study assumes that with the help of a green shared vision by the responsible leaders to protect the environment that are considered to be the supplies by the organization, the followers/employees are in a better position to recognize the need of protecting the environment which also initiate a strong inner aspiration in employees to safeguard the environment and realizing the of environmental responsibility [59]. The current study argues that green shared vision plays a mediating role among the association of responsible leadership and green behaviors. Taking into account the S–V fit theory, which discuss that ecological values provision from the company (e.g. green shared vision) is assumed to be the best fit by the employees who have high level of values to protect environment (individual green values), resultantly they exhibit high level of environmentally friendly behaviors. When the organization fails to give provisions for green values (e.g. green shared vision), it is considered to be a negative factor of an organization by the individuals who have firm eco-friendly beliefs, and these individual react with poor environmental friendly consideration. Hence, with individual green values, the fill will enhance, and substantial existence will increase the green behaviors of employees (in-role and extra-role) (see Fig. 1, Fig. 2).

### 2.3 Hypothesis development

#### 2.3.1 Responsible leadership and green behaviors

At present, the leaders confront a dynamic and complex setting of business that presume them to achieve the financial objectives of the company as well as paying significant consideration to the CSR concerns along with environmental matters [63]. Accordingly, the research [17] put forward the conception of a responsible style of leaders through mixing up leadership with corporate social responsibility. Responsible leaders consider three major elements: effectiveness, ethics, and sustainability. Considering the element of effectiveness, responsible leaders brings encouraging implication for workers and business [64]. Whereas the element of ethics considers that responsible leaders behave ethically and lead by example by acting in the right way and setting an example for their subordinates [38]. While on the other hand, the sustainability element of responsible leadership emphasizes that responsible leadership emphasizes the environmental, social, and financial performance of the business for achieving sustainability and ecological advancement [26]. Responsible leadership positively influences business firms and workers [65]. Considering the green behaviors of employees, is considered a conversation and recycling of resources and includes the behaviors of reducing wastage in the company [65]. The green behaviors of employees include in-role environmental behaviors and extra-role ecological behavior. The green behaviors of workers are potentially influenced by the different contexts of an organization, such as CSR, green HRM as well a responsible style of leadership [66]. Responsible leaders are likely to affect the behaviors of employees by means of informal relations between leader and follower, along with this setup, another action of the organization which impacts the place of work [49]. In addition, a responsible style of leadership can make a transformation in the motivation of workers as well as in their ecological behaviors by the means of informal as well as personal relations between supervisor and subordinate [67]. Responsible leader pays attention to environmental-related and ecological concerns, and they also focus on encouraging workers to exhibit eco-friendly behaviors in the place of work [68]. The supplies and values fit theory claims that the employees of the company value the supplies offered to them [33]. When there is a fit between the organization's supplies and the employees' values, the employees believe that the goal and aims of the organization must be consistent with their objectives [59]. Similarly, under the direction of the responsible style of leadership, the workers are going to recognize the significance of eco-friendly behaviors by learning from their leaders and following them [69], subsequently improving green behaviors) of workers. Considering the above discussion, the potential influence of the responsible style of leaders on in-role environment friendly and extra-role green behaviors has not been examined empirically so far. For this reason, it is argued that responsible leadership influences the green behaviors of employees (in-role and extra-role green behaviors). Hence, the discussed empirical pieces of evidence and theoretical basis, the following hypothesis are proposed:

H1 Responsible leadership positively influences in-role green behaviors of employees.

H2 Responsible leadership positively influences extra-role green behaviors of employees.

#### 2.3.2 Mediation of green shared vision

The concept of green shared vision is known as a strategic, clear, and common direction that focuses on accomplishing the ecological objectives as well as the environmental aspirations of the business firm, which is internalized by the workers [70]. Business firms are greening their work environment by sharing a mutual vision with the workers [22]. Leaders' role in developing the organization's vision is one of the significant contextual elements. The vision of organizations is created by the contribution of the leader and their behavior [23], but the linkage between the green shared vision of the firm and the responsible style of leadership has not been investigated extensively. A responsible leadership style requires that the supervisors or managers be ethically and morally mindful of the internal as well as external stakeholders of the organization. Responsible leaders have been conceptualized and inferred as one of the antecedents of the organizational citizenship behavior of the employees directed towards eco-friendly concerns [71]. Based on this, a responsible leader can pass on signals to their followers their consciousness about social responsibility so that it creates awareness in the employees to behave in a similar responsible way [62] by emphasizing the environmental activities [72]. Additionally, prioritizing the development of workers along with taking into account their long-lasting interests is likely to significantly contribute to employee commitment and engagement, which, as a result, motivates them to accommodate their interests for the sustainability of the organization by means of forfeiting their own welfare and resources as a reaction to the practices of the organization regarding sustainability [19]. Considering the belief that environmentally friendly complications are the outcome of dysfunctional behavior [73], elucidations are associated with the psychology domain along with the tools to change the behavior of individuals [4], particularly the style of leaders [74]. Leaders are the company's representatives, providing direction to employees and appraising them. Furthermore, responsible leaders present a vision for the future created on the ethical orientation that has been imitative from mutual interactions [75]. Moreover, a responsible style of leadership creates an ecological vision as well as an eco-friendly environment for work in the company [48]. With the help of a mutual vision shared by the responsible leaders, the followers are better able to identify the connotation of defending the environs as well as advance a strong inner aspiration to safeguard the environment and encourage the realization of environmental responsibility [50]. According to Ref. [76], to become a self-adapting organization, employee of the company must recognize, shares, and encircle a mutual prophecy. The behavioral response of the followers to hearing about the company vision is crucial since the vision reflects the organization's basic purpose and goal of the organization, which is helpful for the followers to determine which behavior is appropriate, important, irrelevant, or insignificant [77]. Responsible leaders try to regard the environment of the company as one of the main stakeholders [26], which results in advocating for improvements in the natural and work environment, that prompt behavior from the workers that promote a sustainable environment. The responsible style of the leader helps in fostering the green shared vision. Alongside, responsible leadership also emphasizes the connotation of considering the environment [26], and by the means of creating mutual plans for protecting the environment, they promote a green shared vision. When a common vision directs the companies, the employees consider their contributions to the organization meaningful [78]. Therefore, the employees are at ease to exhibit their views about potential improvements in the environment. The research study of [53] identified that the green shared vision is linked with the environmental behaviors of the employees. Along with this, the green shared vision significantly contributes to a firm's innovation performance, whether green incremental or radical [53]. The mutual vision offers the employees a strategic and collective path that guides the workers to use a specific tactic. A common vision of ecological management is critical for improving the green behaviors of employees. According to S–V fit theory [33], when supplies offered by organization are at the same level or exceeds the individual values. There is going to be a best fit which positively influences the behaviors and attitude of individuals. At times, while workers have a high degree of value fit, the behaviors and approaches of the employees towards the company are affirmative [79]. Resultantly, these factors of the organization improve the willingness of the employees to serve their company as well as the organization's goal [80]. The research study [81] argues that the green shared vision of the company improves pro environmental behavior. Considering the discussion, the current study suggests that the relationship between the responsible style of leadership and green behaviors occurs part because of the mediation of green shared vision.

H3 Green shared vision mediates the positive association between responsible leadership and in-role green behaviors.

H4 Green shared vision mediates the positive association between responsible leadership and extra-role green behaviors.

#### 2.3.3 Moderation of individual green values

The literature review on contemporary values assumes that the values of individuals are depicted by their behaviors and attitudes [43]. The research study of numerous research scholar [[82], [83], [84]] claimed that the personal ecological values of the individuals are significantly influencing their ecofriendly behaviors. The common ideology, which is aligning the personal values of the individual with the organizational values increase outcomes of the workers of the organization, such as strengthening the positive behaviors, identification in the organization, and attitudes [85]. When the employees connect themselves with their organization, by aligning their identification and values, the likelihood of the employees committing to achieving the goalmouths of the business is high [30]. As a result, the behavior of employees is an interplay between an employee and the environs [86]. Keeping in view the research of [47], the workers make obvious and clear sentences regarding the behaviors and policies of the company regarding social responsibility, and these judgments determine that the employee's needs are satisfied. Reclaiming from on S–V fit theory [33], when the supplies of organization are conducive to environment to the values of the employees, it will result in congruent of the ecological values of the employees with the values of company, so it is assumed that the probability of employees exhibiting environmental friendly behaviors would be high. On contrary to this, when the values of the employees are not consistent and harmonious with organizational values, or it may be the case that the environment provided by the organization is not matching with the needs of the employees that leads towards employees not demonstrating the ecological behaviors at organization. Scilicet, the environmental values of an individual and the ecological values of the organization interactively impact the ecofriendly actions of the workers in the workplace. The green shared vision reflects the results of the employees' judgments about the organizations green values. The theory of supplies and fit proposes that the provision of an organization with ecofriendly values (e.g., green shared vision) is reflected as the best fit when the employees own a high degree of value for environment (individual green values), and the workers are likely to demonstrate a high degree of ecofriendly behavior. At odds with this, when the organization fails to make provisions of its values (e.g., green shared vision), it is considered to be a negative factor of company by the employees who depicts high level of environmental friendly values, so in return the response of the individuals are poor and they are less concerned about the environment. Subsequently, in the presence of individual green values, the fit among supplies (green shared vision) and value (employee green behaviors) will be best that will increase the employee ecological behavior that includes is in-role as well as extra role. Consequently, the present study perceives that individual green values plays a moderating role among the association of green shared vision and green behaviors of employees. Accordingly, based on the theoretical support and past studies, following hypotheses are postulated:

H5 Individual green values play the role of moderator between the positive association of green shared vision and in-role green behavior.

H6 Individual green values play the role of moderator between the positive association of green shared vision and extra-role green behavior.

## 3 Materials and methods

### 3.1 Procedures, sample, and data collection

The data was collected from the participants using the non-probability convenience sampling due to the less information about population because probability sampling required a list of population [87]. This method works best when the people are diverse and can live anywhere [88]. This method expedited the data collection process, enabling us to promptly gather insights without requiring a substantial financial commitment. As a result, data was gathered from MBA executive program students who had spent at least a year working in a manufacturing organization. The few reasons were to collect the data from MBA executive programs. Having deep insight into the manufacturing process helps to collect data and consider suitable respondents. Secondly, since the English language is well thought-out to be the official language of Pakistan [89], most employees lack the necessary education to complete the questionnaire [90], that is why the questionnaire was only given to educated respondents [91]. Second, it cannot be easy to obtain permission from managers to collect data at work [92]. This could be because data collection takes a long time and prevents employees from doing their jobs. Finally, employees are not at ease discussing their bosses at work.

We collected data using a survey questionnaire, a well-established and reputable instrument in the field of social science research. This method is known for its reliability in gathering information and viewpoints on prevalent attitudes and behaviors among individuals [93]. Using a questionnaire based on the survey method, 450 questionnaires were distributed, but only 307 valid responses were returned. To determine the sample size, a 20 to 1 ratio has been suggested by Ref. [94]. This study has 21 items, and according to the above described method, our minimum sample size was 420. To adhere to ethical standards, the researcher assured respondents that their responses would be kept confidential [95]. The researchers took precautions to avoid the problem of common method variance because the study measured explained and explanatory variables simultaneously [96].

### 3.2 Measurements of variables

The survey questionnaire of the present study has been adapted from the previous studies, and all the constructs were tested previously. The respondents were asked to provide their response using the scales of responsible leadership, green behaviors (in-role green behaviors and extra-role green behaviors), green shared vision, and individual green values, having a “five-point Likert scale ranging between 1-strongly disagree to 5-strongly agree”.

#### 3.2.1 Responsible leadership

The variable of responsible leadership was measured by adapting the scale developed by Ref. [97]. The scale has five (05) items and a sample item of the scale: “My immediate supervisor demonstrates awareness of the relevant stakeholder claims.” The reliability of the scale was 0.914.

#### 3.2.2 Green shared vision

To measure the green shared vision, the scale of [21] was used, having four (04) items in the construct. The item reliability of items on the green shared vision scale was 0.897. The item example of green shared vision consists of “A commonality of environmental goals exists in the company”.

#### 3.2.3 In-role and extra-role green behaviors

For measuring the green behaviors [43], adopted the scale of in-role and extra-role green behavior of [47]. The scale included a total of six (06) items, three (03) items were for in-role green behaviors, and three items were for extra-role green behaviors, with the reliability of the scale as 0.86 and 0.85, respectively. The sample item of in-role green behavior was “Today, I performed tasks that are expected of me in environmentally friendly ways,” A sample example of extra-role green behaviors includes “Today, I took a chance to get actively involved in environmental protection at work.”

#### 3.2.4 Individual green values

To measure individual green values, the scale of [96] was adapted. The scale has six (06) items showing a reliability value of 0.84. The sample scale item was “I feel a personal obligation to do whatever I can to prevent environmental degradation.”

#### 3.2.5 Control variables

In the current study, age, education, work experience, and gender are used as control variables. These variables may affect the perception of green behavior [97], ethical leadership [98], individual green values [99] and green human resource management [100].

## 4 Results

### 4.1 Preliminary analysis

Before putting the hypotheses to the test, the researchers looked for missing values, outliers, and problems with normality and correlation [101]. 17 careless or incomplete responses were assumed to be redundant during the second data collection phase. This meant that 307 final answers did not have a missing value. Both the values of skewness and kurtosis were falling in the acceptable ranges (between 3 and 1), which shows that the data were evenly spread out [102].

Herman single factor test was performed to check the common method variance. The results show that the first component has an initial eigenvalue of 9.150, which explains 43.572 % of the variance in the data, which is less than 50 % which indicate that there is no issue of biases and can go for further analysis. The value of the correlation between constructs is also below 0.90, signifying the nonexistence of the issue of common method variance [94].

### 4.2 Reliability and correlation analysis

Table 2 represents the reliability and correlation analysis. Cronbach's alpha coefficient of 0.70 or higher is acceptable for research purposes, indicating that the items in a scale or measure are reliable and consistently measure the same underlying construct. All variables exhibited high levels of internal consistency reliability, with Cronbach's alpha coefficients ranging from 0.703 to 0.937. The variable with the highest reliability was responsible leadership (α = 0.937), followed by in-role green behavior (α = 0.866), green shared value (α = 0.811), extra-role green behavior (α = 0.790), and individual green values (α = 0.703). These coefficients indicate that the items measuring each variable were highly reliable and consistent in measuring the same construct.

**Table 2:** Reliability and correlation analysis.

Variables	Cronbach Alpha	1	2	3	4	5
Responsible leadership(RL)	0.937	1				
Green Shared Value(GSV)	0.811	0.574**	1			
In role Green Behavior(INGB)	0.866	0.446**	0470**	1		
Extra Role of Green Behavior(EXGB)	0.790	0.360**	0.396**	0.678**	1	
Individual Green Values(IGV)	0.703	0.450**	0.289**	0.429**	0.421**	1

**p < 0.05.

Table 2 shows the correlation coefficients among five variables: responsible leadership, green shared value, in-role green behavior, extra-role green behavior, and individual green values. The correlation coefficients range from 0 to 1 and represent the strength and direction of the linear relationship between two variables.

According to Table 2, responsible leadership is significantly correlated with other variables. This suggests that being a responsible leader necessarily leads to more green behavior or values among employees. There are significant positive correlations among the other variables. Green shared value is positively correlated with in-role green behavior (r = 0.574, p < 0.01), suggesting that employees who place more value on environmental sustainability are more likely to engage in environmentally responsible behavior required by their jobs. In-role green behavior is also positively correlated with extra-role green behavior (r = 0.678, p < 0.01), indicating that employees who engage in more environmentally responsible behavior at work are also more likely to engage in such behavior outside work. Individual green values are positively correlated with both in-role (r = 0.446, p < 0.01) and extra-role (r = 0.396, p < 0.01) green behavior, suggesting that employees who hold stronger environmental values are more likely to engage in environmentally responsible behavior both within and outside of their work roles.

### 4.3 Measurement model

#### 4.3.1 Statistics of confirmatory factor analysis

Table 3 depicts factor loadings, composite reliability (CR), and average variance extracted (AVE) of the five variables (EXGB, INGV, RL, GSV, and INGB) were examined by structural equation modeling in AMOS. The results signpost that all variables had strong factor loadings ranging from 0.770 to 0.922 for RL, 0.791 to 0.793 for EXGB, 0.818 to 0.842 for INGV and INGB, and 0.876 to 0.892 for GSV. The CR values ranged from 0.818 to 0.937, indicating good internal consistency. The AVE values ranged from 0.591 to 0.788, meaning that each variable accounted for a moderate to a high proportion of its variance. The model fitness indices indicated a good fit to the data, with a chi-square to degrees of freedom ratio (CMIN/DF) of 2.851, a comparative fit index (CFI) of 0.935, a goodness of fit index (GFI) of 0.876, and a root mean square error of approximation (RMSEA) of 0.078. These results suggest that the model adequately represented the data and that the variables included in the model were well-defined and distinct from one another [103].

**Table 3:** Model fitness indicators.

Variables	Factor loadings Range	CR	AVE
EXGB	0.791–0.793	0.818	0.600
INGV	0.818–0.842	0.896	0.591
RL	0.770–0.922	0.937	0.750
GSV	0.876–0.892	0.918	0.788
INGB	0.818–0.842	0.868	0.687
Model Fitness indices: CMIN/DF = 2.851, CFI = 0.935, GFI = 0.876,RMSEA = 0.078			

RL = Responsible Leadership, INGB= In Role Green Behavior, EXGB = Extra Role Green

Behavior, GSV = Green Shared Value, INGV= Individual Green Values

### 4.4 Discriminant validity

The diagonal values represent the discriminant validity or the extent to which each variable (represented by the rows and columns) measures a unique construct or factor different from the other variables. These values are relatively high, indicating that each variable measures a unique construct that is not highly correlated with the other variables (see Table 4). These results suggest that the variables of current research have good discriminant validity and can be considered distinct and independent measures of the constructs they represent.

**Table 4:** Discriminant validity.

	EXGB	INGV	RL	GSV	INGB
EXGB	0.775^a^				
INGV	0.642	0.769^a^			
RL	0.500	0.621	0.866^a^		
GSV	0.329	0.438	0.545	0.888^a^	
INGB	0.524	0.505	0.488	0.316	0.829^a^

Note(s): a AVE square root in diagonal, RL = Responsible Leadership, INGB= In Role.

Green Behavior, EXGB = Extra Role Green Behavior, GSV = Green Shared Value,

INGV= Individual Green Values

### 4.5 Regression results

Table 5 depicts the estimated path coefficients. First, the direct effect from RL to INGB (β = 0.391, p < 0.01) and RL to EXGB (β = 0.467, p < 0.01) are found significant. When leaders prioritize and adopt responsible and sustainable practices, it encourages their team members to adopt green behaviors while performing their job duties, as well as outside their job duties. The first 2 direct hypotheses are found significantly evident.

**Table 5:** Hypotheses results.

	Estimates	CR	P VALUE
RL→ INGB	0.391	7.839	***
RL→ EXGB	0.467	7.426	***
Note: ***p < 0.001			
**Mediation analysis**			
	**Direct B W/O Mediation**	**Direct B With Mediation**	**Indirect Effect**
RL→ GSV→ INGB	0.115***	0.148***	0.146***
RL→ GSV→ EXGB	0.434***	0.448***	0.046^(NS)^
Note: Confidence interval, bootstrap sample size 5000			
**Moderation analysis**			
	**Estimates**	**CR**	**P VALUE**
GSV × INGV→ INGB	−0.070	−2.555	0.011
GSV × INGV→ EXGB	−0.095	−14.431	***

Note: RL = Responsible Leadership, INGB= In Role Green Behavior, EXGB = Extra Role Green Behavior.

GSV = Green Shared Value, INGV= Individual Green Values.

Moreover, the results for the control variables show a non-significant effect on in-role green behavior: gender (β = 0.280, p = 0.77), age (β = −0.007, p = 0.872), education (β = −0.154, p = 0.232), and experience (β = 0.041, p = 0.478) as well as on extra role green behavior: gender (β = 0.157, p = 0.422), age (β = −0.003, p = 0.957), education (β = 0.062, p = 0.696), and experience (β = 0.026, p = 0.720).

For mediation analysis, the bootstrapping technique is used to assess the indirect effect of green shared value as recommended by Ref. [104]. The direct beta without mediation is 0.115, and this effect is statistically significant at the 0.001 level. The direct beta with mediation is 0.148, and this effect is also statistically significant at the 0.001 level. The Indirect beta is 0.146, which is statistically significant at 0.001. Green shared value has been seen as mediated between RL and INGB. Thus, H3 is supported. While checking the mediating effect of a green shared vision between RL and EXGB, the direct beta without mediation and direct beta with mediation are 0.434,0.448, respectively, and statistically significant at the 0.001 level. However, the indirect effect is 0.046, which is not statistically significant. Therefore, H3 is not supported as the indirect effect has been seen as insignificant.

For moderation analysis, the interaction between GSV and INGV was computed to see the significant effects on both types of green behavior. The p-value for INGB is 0.011, below the conventional threshold for statistical significance (i.e., 0.05). The p-value for EXGB is reported as "***", indicating a highly significant effect. The results suggest that the association between green shared vision and green behavior (in role and extra role) is moderated by individual green behavior. H5 and H6 are supported as suggested in the literature.

## 5 Discussion and conclusion

The role played by responsible leaders(RL) to promote ecological behaviors within organizations cannot be denied. According to Ref. [105], RL is conscious of the influence of their activities on the environment by promoting green behavior and initiatives among employees. This study aimed to investigate the impact of RL on employees' in-role and extra-role green behaviors, the mediating role of green shared vision(GSV), and individual green values(IGV) as moderator. The first hypothesis is related to RL, which positively influences in-role green behaviors of employees(H1). The results of the study support this hypothesis, showing a significant positive relationship between RL and employees' in-role green behaviors. The results are consistent with prior research that has found that RL positively impacts the green behavior of workers [66]. The S–V fit theory [33] suggests that while there is a congruence between the values of the individual and the organization, the employee is more likely to be committed to the organization and perform better. In the context of this study, employees who share similar environmental values with their organization are more likely to engage in green behaviors. Thus, the S–V fit theory can explain the positive relationship between RL and green behaviors, as RL can help create an organizational culture that aligns with employees' environmental values. Secondly, this study developed hypothesis which suggested that RL positively influences extra-role green behaviors of employees (H2). The result of the study also supports this hypothesis, showing a significant positive relationship between RL and employees' extra-role green behaviors. The outcomes of current study are consistent with past studies which examined that RL positively impacts employees' extra-role green behaviors [68].

This study checks the mediation of GSV among RL and the role of green behavior of employees(H3). The result supported our hypothesis, and the finding of this study is similar to a previous study [106]. By fostering a shared environmental sustainability vision, RL can create a culture that values and prioritizes pro-environmental behavior. Our findings extend the supplies-values fit (S–V fit) theory, which argues that the supplies provided by the RL, the employees behave accordingly (green behavior).

Furthermore, the study examines that RL causes the GSV that ultimately enhances extra role green behavior (i.e., H4). However, the result is not consistent with previous studies. One possible explanation for the lack of mediation is that the relationship between RL and extra-role green behavior is more direct than the relationship between RL and GSV. RL may influence extra-role green behavior by setting environmental goals, providing feedback and incentives, and modeling environmentally responsible behavior. These actions may be more tangible and observable to employees than the shared vision of environmental sustainability. Another possible explanation is that GSV may be more closely linked to in-role green behavior, which is part of job responsibilities, rather than extra-role green behavior, which refers to voluntary pro-environmental actions. Extra role green behavior might be influenced by other variables like green human resource management and ethical leadership [96].

Finally, this study checks the moderating role of INGV in the relationship between GSV and in role and extra role green behavior (i.e., H5 and H6). The results revealed that INGV significantly moderated in both relationships and supported our hypotheses. The results are also novel as, to the best of our knowledge, no study has yet investigated the moderating role of individual green values between GSV and in role and extra role green behavior. The supplies-values fit (S–V fit) theory posits that for an organization to effectively embrace ecofriendly values, such as a green shared vision of environmental sustainability, it is most successful when its employees hold a strong personal regard for the environment (individual green values). In such cases, employees are more inclined to exhibit a significant environmental friendly behavior. Employees with strong individual green values will likely be motivated to engage in environmentally responsible behavior. Therefore, the effectiveness of the green shared vision in promoting employee green behavior, including in-role and extra-role green, may depend on the extent to which individual employees hold these values [107]. This highlights the importance of understanding differences in the values of individuals and how they may intermingle with organizational initiatives in promoting environmentally responsible behavior [107]. Leaders must recognize and consider these individual differences while designing and implementing sustainability initiatives in their organizations.

## 6 Implications

### 6.1 Theoretical implications

The prevailing research will extend the literature on the organization's environmentally friendly sustainability differently. Past research studies indicated that leaders play a crucial part in predicting the ecological behaviors of the employees at the workplace. Astoundingly, it was known very little about the effect of responsible leadership, emphasizing ecological sustainability, which is likely to shape the behaviors of employees in the workplace, as the role of leaders in predicting green ecological consequences is rare. Relying on Supply-Value fit theory, the research proposes that the firm offers responsible leadership to employees. In return, the employee values the supplies by depicting ecofriendly behaviors. Employees will likely imitate the leaders' behavior and improve the environment. The ecofriendly behaviors of employees are beneficial for the environs as well as for the organization. The study provides the approach by analyzing the processes of green shared vision, which is triggering the eco-friendly behaviors of workers. It is one of the important implications of the study that can be helpful for the organization to reach its environmental goals and attain sustainable development. The study will contribute to the literature of responsible leaders as the research conducted in past was mainly concentrating on the theoretic evaluation of responsible leadership, while the empirical researches based on the approach of theory-orientation was comparatively meagre. The study clarifies the underlying mechanism through which responsible leaders are likely to promote the green behaviors of employees, indicating a different role by analyzing in parallel the indirect effect of the green shared vision. The results highlighted that the mediating role of a green shared vision among responsible leadership and employee in-role green behaviors is positive, whereas, with extra-role green behavior, the relation is not significant. The inconsistent findings recommended that the introduction of a moderator may impact the influences. So, individual green values serve as a conditional factor among the relationships. The study suggests that responsible leadership is a strategic resource of business that coordinates the green shared vision of the company to influence employee ecological behaviors by moderating the effect of individual green values. When the individual has green values, they are more likely to exhibit green behaviors than employees who lack green values.

### 6.2 Practical implications

The research indicates that responsible leadership is one of the essential triggers of ecological behaviors in the workplace. For this reason, the organizations must give extra attention to selecting, appraising, and advancing the leaders that can cultivate the skills and abilities of responsible leaders. The international service-learning program indicated that a responsible leader must have a series of capabilities, including culture-based intellect, ethics-related literacy, a responsible approach, and a social development perspective. Subsequently, the firms should consider the socially responsible actions of the leaders. Consequently, the firm should consider its green shared vision that includes the ecological behaviors of the employees while they work. Responsible leadership can transfer signals to the firm about its sustainable and ecological beliefs and values to improve the employees' awareness level about social responsibility and ethical concerns, which influence the ecological practices and behaviors of the workers. Hence, improving the supervisor's capabilities of responsible leaders and strengthening the interaction of managers with subordinates are advantageous to encourage the protection practices of environment at the workplace. The human resources practices of an organization should also focus on reflecting a desire for recruiting and hiring candidates who demonstrate the characteristics and norms of being a responsible leader. To strengthen the green behaviors of employees, it should be ensured that the managers are selected, promoted, trained, and developed according to responsible leaders' values as employees intimate the behaviors of their leaders. Business organizations are focusing on greening their setting and situations through a green shared vision that is a mutual vision of the company and its workers. The leader's role is a substantial contextual factor in developing the firm's vision. To develop the firm's vision, the leaders' behavior contributes together. The association of responsible leaders and green employee behavior with the indirect path of the green shared vision has been examined parochially. Moreover, there is a need to focus on creating the organizations' culture based on a green shared vision, as the organization's green shared vision is important in stimulating the ecological behaviors of employees through enacting the policies and practices related to environment.

## 7 Limitations and future research directions

### 7.1 Limitations

The present study has some limitations that should be addressed in future research. This study is cross-sectional; thus, causality cannot be established. This study relied on self-reported green behavior measures, which may be subject to social desirability bias. This study considers a single industry, and thus, the generalizability of the findings may be limited. One limitation of this study is that the data collected were self-reported by the employees, which may introduce response bias and limit the reliability of the results. Alternative methodologies could be explored to mitigate this limitation and enhance the robustness of the findings. Incorporating objective measures or third-party assessments could provide a more comprehensive view of the variables under investigation. This approach could help reduce the influence of response bias and enhance the reliability of the results. It is important to recognize that this study was conducted within a specific culture. Other cultural factors can significantly impact individuals' behaviors. Therefore, the findings of this study may not necessarily generalize to other cultures. Conducting similar research in other cultural contexts can gain valuable insights. This study is quantitative; further qualitative or mixed method research can be conducted. It is not possible to include all the variables in one study; other variables might influence the relationship.

### 7.2 Future research directions

Future research should use longitudinal designs to examine the causal relationship between responsible leadership and employees' green behaviors. Future research should use objective measures of green behavior, such as energy consumption data. Future research should replicate the study in different sectors. To overcome the limitations of the study, future research could use multiple data sources, such as supervisor ratings or objective measures, to provide a more comprehensive and accurate assessment of employees' green behaviors. Another possible future direction could be investigating the mediating mechanisms between responsible leadership and employees' green behaviors using the S–V fit theory [33]. Our research investigates the mediating role of green shared vision in the link between responsible leadership and green behavior. Future research could consider alternative intermediary variables, such as environmental awareness, the ecological climate, and employees' goal orientation [106]. This theory also suggests that individuals are more likely to engage in behaviors that align with their values. Thus, responsible leadership may influence employees' green behaviors by promoting a values-based culture that prioritizes sustainability. Responsible leadership is a relatively new concept; its impact on other employee behaviors like organizational citizenship behavior, task performance, and innovative work behavior should be explored in future research. Future research could explore the part of individual values and values congruence in the association between responsible leadership and employees' green behaviors.

## 8 Conclusion

The focus on employees' green behaviors as a significant aspect of achieving organizational sustainability has gained considerable attention from both scholars and practitioners. Within this domain of research, our efforts have been dedicated to explain the mechanisms that foster employees' green behavior. To address this, we have adopted the Social-Values Fit (S–V fit) theory as a foundational framework for the development of our conceptual research model. This model incorporates key variables that serve as antecedents to employees' green behaviors including responsible leader, green shared vision and individual green values. The study found that the more responsible leadership will be, the greener behavior will be in the employees. Secondly, mediation effect of green shared vision mediates between responsible leadership and in role green behavior. While it has also seen that green shared vision does not mediates between responsible leadership and extra role green behavior. Testing the moderation mechanism of green values individuals on a green shared vision and green behavior revealed that INGV significantly moderated in both relationships.

## Data availability statement

Data will be made available on request.

## Funding

This research received no external funding.

## CRediT authorship contribution statement

**Nimra Younas:** Conceptualization. **Md Billal Hossain:** Validation. **Aleena Syed:** Validation. **Sarmad Ejaz:** Software, Conceptualization. **Faisal Ejaz:** Methodology. **Tahir Saeed Jagirani:** Validation. **Anna Dunay:** Resources.

## Declaration of competing interest

The authors declare that they have no known competing financial interests or personal relationships that could have appeared to influence the work reported in this paper.
